# A Comparison of Chinese and Korean Older Adult Immigrants’ Transnational Healthcare Practices in Toronto, Canada: A Mixed-Methods Study

**DOI:** 10.3390/healthcare13192493

**Published:** 2025-10-01

**Authors:** Leah Czukar, Lu Wang, Sepali Guruge, Janet Lum, Meira Greenbaum

**Affiliations:** 1 Department of Geography and Environmental Studies, Toronto Metropolitan University, Toronto, ON M5B 2K3, Canadameira.greenbaum@torontomu.ca (M.G.); 2 Faculty of Community Services, Toronto Metropolitan University, Toronto, ON M5B 2K3, Canada; sguruge@torontomu.ca; 3 Department of Politics and Public Administration, Toronto Metropolitan University, Toronto, ON M5B 2K3, Canada; jlum@torontomu.ca

**Keywords:** older adults, transnational health practices, Chinese immigrants, Korean immigrants, healthcare access barriers, THP, Canada

## Abstract

Background/Objectives: While immigrants represent 21% of Canada’s total population, they represent 30% of the country’s older population. Sociocultural and economic barriers to the Canadian healthcare system have been frequently reported among older adult immigrants. These barriers are intricately linked to a vastly understudied phenomenon-transnational health practices (THP), which may involve travelling to home countries for healthcare, accessing medicine and health-related information and resources linked to home countries. This study aimed to explore the relationships among local healthcare experiences in Canada, individual characteristics and use of THP among older adult immigrants. Methods: A mixed-methods approach was used combining statistical, spatial and qualitative methods to analyze group patterns of THP and its influencing factors. Primary data was collected through surveys and focus groups of older Mainland Chinese and older South Korean immigrants residing in Toronto. They are the two largest East Asian groups in Canada, with documented transnational ties with their home country. Results: The study found that THP were sought by both groups but were more prevalent among older Chinese immigrants. By integrating quantitative and qualitative analyses, the study revealed complex relationships between THP and barriers in local healthcare access relating to wait times, cost, language, availability, spatial accessibility and quality of care, for different types of care including primary, specialist, eye and dental care. Conclusions: The study generates new knowledge on THP in Canada and adds to the growing body of literature on transnational healthcare practices and behaviours among migrants across different countries and regions. It provides implications to inform health policy and deliver care for older adult immigrants as their populations continue to increase.

## 1. Introduction

Canada’s publicly funded universal healthcare system is a point of national pride. However, is it meeting the challenge of a rapidly aging and increasing diverse population? While the immigrant population accounts for about 23% of the Canadian population, immigrants make up 30% of individuals who are 65 years or older [1]. Older adults tend to rely heavily on healthcare systems, accounting for 46% of national public health expenditures in Canada [2]. According to the Canadian Institute for Health Information’s (CIHI) Commonwealth Fund Survey (CMWF), Canada ranks the lowest for access to primary care among 10 comparable countries, such as the United Kingdom and Australia [3]. More specifically, 86% of the Canadian population has access to primary care as compared to the 93% CMFW average. Older adults face challenges related to health problems associated with aging, as well as long wait times, travel distance and mobility issues [4,5]. Older adult immigrants in Canada often experience additional barriers in accessing care due to their limited proficiency in English, declining spatial mobility, cultural differences, lower socioeconomic status, and difficulty navigating the Canadian healthcare system [4,6,7,8,9].

To mitigate challenges in accessing healthcare in a destination country such as Canada, older adult immigrants have been found to follow transnational healthcare practices (THP) [10,11,12], which can be defined as a range of practices used by immigrants seeking health-related information and resources from their home country [13,14]. THP involves a range of health-related behaviours, including accessing home country-based medicine, seeking healthcare information online from home country-based websites (non-travel-based THP), or travelling to the country of origin to receive healthcare services or products (travel-based THP) [15]. Motivations for THP include better affordability, familiarity and availability of culturally responsive and linguistically appropriate services [13,16]. Much of the current THP research focuses on Latin-American immigrants in the U.S. seeking healthcare from their home countries, such as Mexico [16,17,18,19], and transnational care use among migrants in European countries and Turkey [10,20,21]. Canadian studies of THP remain limited. Based on the 2021 Census of Population by Statistics Canada, Asia accounted for 62% of recent immigrants to Canada. Current research on Asian immigrants’ experiences in accessing THP is limited, and among the four studies identified [11,13,22,23], only one study [13] specifically focused on older adults in Canada.

This study aims to examine the patterns of THP among older adult immigrants in the Toronto Census Metropolitan Area (CMA) and factors related to individual socioeconomic status, quality of local healthcare received, spatial access to local health care and transnational ties. The study focuses on older adults from South Korean and Mainland Chinese communities, the two largest East Asian populations in Canada. In this paper, South Korean immigrants are referred to as Korean immigrants, and Mainland Chinese as Chinese immigrants. A comparison between the two groups reveals culture-specific THP patterns and the variation of local healthcare experiences for different immigrant groups. The paper employs a mixed-methods approach that integrates spatial-quantitative analysis of survey data and qualitative analysis of focus group (FG) data.

## 2. Scholarly and Conceptual Background

This study is situated in the broad phenomenon of transnationalism and the emerging literature on how immigrants navigate their access to healthcare simultaneously both locally in host country and transnationally in country of origin. Transnationalism refers to the social, economic, and political processes occurring beyond national borders [15]. Under the concept of transnationalism, immigrants are viewed as actively promoting cultural changes in places of origin and destination, which varies from the assumptions of immigrant assimilation to a destination country [15,24,25]. Transnational social ties to relatives and friends from immigrants’ home country greatly influence the connection between places, as Madianou [26] defines as “co-presence” between countries of origin and destination. Specifically, transnationalism has been found to influence health knowledge and attitudes, healthcare access and health outcomes among immigrant populations [27]. Immigrants’ transnational social networks are important for meeting their healthcare needs in both destination and origin countries [28,29]. Analyzing health needs of immigrants in a transnational context provides greater insights into how older adult immigrants navigate healthcare in their destination country.

Travel-based THP requires a level of mobility since immigrants must physically return to their home country to receive healthcare services [15], while non-travel-based THP is often used by immigrants with limited travel access and involves utilizing health-related information (online or phone based) and health products (such as medicine) linked to their home country [15,17,19]. In some cases, travel-based THP is a secondary reason for travel, with the priority of travel being visiting family or friends, and then receiving healthcare while already in the home country [20]. In other cases, THP is the primary reason for travel, as a response to familiar and culturally similar general practitioners (GPs) or specialists [22]. Travel to the home country for healthcare often results from an individual’s preference for home country healthcare [30]. THP has been observed among diverse migrant groups including documented migrants, immigrants who maintain frequent transnational ties [15,17,19,20], undocumented migrants who may have limited access to health services due to a lack of insurance in the destination country [31] and refugees who may or may not maintain transnational connections due to their refugee status [32].

In the host country, cost and insurance have been found to contribute to THP for transnational migrants. Access to lower cost healthcare has been found to influence Mexican migrants in the U.S. utilizing Mexican healthcare [17] and Turkish migrants in Western European countries accessing healthcare in Turkey [20]. Furthermore, THP can be facilitated by having access to certain health insurance in the home country, such as government insurance schemes or the urban resident basic medical insurance (BMI) scheme for Chinese immigrants living overseas [11]. China’s BMI is part of a national healthcare system that follows a multi-level structure, where over 95% of Chinese citizens are covered by the BMI which aids in covering healthcare costs [33]. While basic public healthcare is subsidized, there are also many privatized options to care such as a rapidly growing private hospital market [34]. South Korea’s National Health Insurance (NHI) system subsidizes user’s healthcare costs, but also requires out-of-pocket payments [35]. Canada has a public healthcare system, based on one of the 5 guiding principles of the Canadian Health Act that healthcare must be universal to ensure equal access to care for all Canadians [36]. However, province-based insurance plans do not cover certain expenses such as dental or vision care [36], and other drawbacks relating to barriers with the accessibility of the Canadian public healthcare system exist [4,5].

Older adult immigrants with strong preferences for their home country healthcare system are found to exhibit a clear inclination toward THP. For example, first generation South Korean adults aged 40–55 in New Zealand have a favourable attitude towards the Korean health system due to their cultural ideas of healthcare standards [22]. Similarly, Chinese older adult immigrants in New Zealand are engaged in THP because they were unaccustomed to some of the western medical practices and preferred their home country’s healthcare standards [11]. THP can signal a lack of quality care and potential health inequities for immigrants in destination countries [15]. Older adult immigrants with limited knowledge of the Canadian official languages are more likely to receive subpar healthcare in Canada and may experience difficulties accessing the healthcare system [37,38]. Those seeking care transnationally experience easier communication with practitioners from their home countries, resulting in better understanding of medical terms and diagnoses [8,11,22]. Long wait times for specialist care and receiving test results are also major barriers in receiving quality health care across all populations in their destination country, which often have publicly funded healthcare [11,13,20,22,39,40]. Availability and wait time barriers present in immigrants’ destination countries can frequently be avoided in South Korea and Mainland China because of privatized physician clinics and hospitals [11,22]. Due to the limited Canadian literature on THP, the impact of these intersecting spatial, socioeconomic, cultural, and transnational factors on THP remains an area for in-depth investigation.

## 3. Study Area and Study Population

The study focuses on Chinese and Korean older adult immigrants residing in the Toronto Area in Canada. The Toronto Census Metropolitan Area (CMA) has a highly diverse population, where racialized populations make up 57% of the total population [1]. Mainland Chinese and South Korean are the two largest East Asian populations accounting for approximately 10.8% and 1.2% of the total population of the Toronto CMA [1]. Comparatively, Mainland Chinese and South Korean immigrants account for 2% (715,835) and 0.4% (138,355) of the total Canadian population [1]. The mid-1990s saw an increase of both Chinese and Korean business and economic class immigrants into Canada, with higher education and economic levels [41,42]. About 60% of Chinese immigrants from 1990s–2009 were from middle class higher-paying occupational groups [41]. Korean immigrants arriving during this period also had high levels of education, relevant work experience and/or financial assets [13]. Strong transnational ties with their home country have been documented for both groups. For example, Chinese immigrants resorted to transnational resources, such as home country-based social network, virtual community events, familial ties and healthcare, to overcome various integration-related barriers [12,43]. Korean immigrants in Canada have also been observed to sustain tight transnational contacts with families and friends in South Korea through a variety of cultural and economic activities [44] and engage in THP [13].

## 4. Data

Primary data for the study were collected from surveys and FGs during a time spanning different phases of the COVID 19 pandemic from October 2021 to March 2023. Ethics approval was obtained through the Research Ethics Board (REB 2021-192) at the authors’ institution. All the surveys and FGs were conducted in the preferred languages of the participants-Mandarin Chinese for Chinese participants and Korean for South Korean participants, recorded and translated into English and transcribed for analysis. Surveys on Google Forms and by telephone were administered to Chinese participants due to COVID-19 social distancing restrictions at the time of the survey, while in-person survey was conducted among Korean participants after the restrictions were lifted gradually. A snowball sampling strategy was used in recruiting participants meeting the following criteria: 55 years of age or older; self-identify as Chinese or South Korean and born outside of Canada; have lived in Canada for more than six months; can understand and consent to participation; live at home (NOT living in nursing or long-term care home); reside in the Toronto CMA; and have an Ontario Health Insurance Plan (OHIP). While 65 and older remains a key marker for benefits and support systems in Canada, the term older adult may be used more broadly to refer to older individuals. Since many older immigrants in Canada migrate from developing nations, a 55-and older cut-off used to define older adults is deemed appropriate to reflect cultural differences in viewing aging populations [4,45]. The surveys collected quantitative information on demographic characteristics, locations of participants and their General Practitioners (GPs), and local and transnational healthcare utilization patterns. Surveys that were duplicates or were of poor quality (e.g., blank answers to key questions) were removed from the original sample size of 201, which included 96 Chinese participants and 105 Korean participants. After survey elimination, the final sample sizes used in this study are 77 for Chinese, 84 for Korean and a total combined sample of 161. Supplementary quantitative data used in our study includes information on GP location compiled from the website of College of Physicians and Surgeons of Ontario [46], and geo-referenced census data on Chinese and Korean population at a census tract level [47]. For each study group, four FGs of 21 Chinese and four FGs of 25 Korean participants were conducted to explore in-depth lived experiences and views of THP and healthcare experiences in Canada. FG participants also filled in the same survey, representing a subset (of the survey sample) that provides both quantitative and qualitative data. Similar to the surveys, FGs were conducted with Chinese participants on Zoom and FGs with Korean participants were conducted in-person following public health guidelines during different phases of the COVID-19 pandemic.

## 5. Methods

This study follows a mixed-methods approach characterized by a Partially Mixed Concurrent Dominant Status Design [48]. Survey-based quantitative and FG-based qualitative research occurred within approximately the same time frame, with a greater emphasis on quantitative analysis. Quantitative analyses include two binary logistic regression models for the combined sample that analyzes factors associated with THP. Logistic regression models are used due to the dichotomous nature of the dependent variables [49], which refer to presence (or non-presence) of THP. Specifically, Model 1 explores predictors associated with non-travel-based THP based on the survey question “Do you keep medicine from your home country, for example, medicine brought to Canada in person, by others, or by mail?” (Answer yes/no). Here, medicine is defined broadly in the survey including cultural medicine, herbal supplements, over-the-counter medications, or other forms of prescription and non-prescription medicines. Model 2 focuses on travel-based THP based on the question “Since you came to Canada, have you travelled back to your home country and received healthcare services?” (Answer yes/no). Independent variables are derived from survey questions on demographic, local healthcare utilization and transnational ties. One additional independent variable measures spatial accessibility to same-language general practitioners (SLGP) at a census tract level, using data on GP location and self-reported languages [46] and geo-referenced census data on Chinese and Korean immigrant population at a census tract level [1]. Spatial accessibility represents simple GP-to-population ratios using a 5 km radius, standardized using z scores representing “high”, “average” “low” and “very low” levels of spatial access to SLGP, following a method suggested by Wang and Ramroop [50]. All independent variables were coded categorically and went through preliminary significance testing using Chi-square and univariate regression analysis. Chi-square statistics are ideal for identifying suitable independent variables measured at a nominal level [51], while univariate analysis was used to confirm the significance of the identified variables, another method for determining independent variables for logistic regression models [52]. A linear regression model was then performed to check for multicollinearity between the selected independent variables. If VIF values were an acceptable score of below 10, the independent variable was included in the analysis. Final independent variables were entered into the logistic regression Models 1 and 2 (see Appendix A for original survey questions to the variables).

Qualitative analysis of FG data involves variations of formulating codes and themes to aid in understanding group experiences [48]. Analysis of the FG transcripts involved reading transcripts back-to-front, identifying questions asked by the FG moderators under two themes: participants’ local healthcare and THP experiences, and using codes or ‘keywords’ to identify sub themes among participant answers to these questions. Keywords or ‘codes’ in the answers to FG questions may have included ‘medication’ to identify experiences using non-travel-based THP, or ‘wait time’ for local healthcare experiences. Some sub-themes include views of and experiences in using primary healthcare in Canada, and whether and how participants had sought or received health care services when visiting their home country. All local healthcare experiences identified through this process are examined in relation to THP using participant quotes on how local healthcare experiences influenced their THP.

Using the mixed-methods framework proposed by Wang [53], cross-tabulation analysis was conducted to link key quantitative and qualitative findings by FG participants. Significant independent variables (e.g., factors and barriers influencing local healthcare, frequency and nature of transnational ties), and dependent variables (travel-based THP, non-travel-based THP) from the logistic regression models are cross tabulated by all FG participants with quotes on their local health care and transnational healthcare experiences extracted from qualitative analysis. A subset of these FG participants with diverse age and gender groups, and varying levels of travel-based and non-travel based THP use were then selected from each group (Chinese, Korean) for an in-depth analysis of healthcare experiences and THP. This method helped to understand how predictors of THP align with potential patterns of dis(satisfaction) with Canadian healthcare, THP experience (e.g., satisfaction with transnational care) and forms of THP (e.g., medication for certain conditions brought from home country) sought by Chinese and Korean participants.

## 6. Results

### 6.1. Quantitative Results from Logistic Regressions

Table 1 reports the descriptive statistics of the key demographic and THP of survey participants. Chinese and Korean sub-samples are similar in many dimensions such as age structure, gender, and employment status. The majority were women, between 65 to 84, and retired or non-working, which includes any unpaid labour, such as volunteering, housework, and taking care of grandchildren. A few group differences are apparent. More Korean participants were long-standing immigrants with 85.7% coming to Canada before 2000, compared to 36.4% of Chinese participants. Their income is higher with 70.2% earning more than $30,000 a year, compared to 41.6% for Chinese participants. For non-travel-based THP, only 9.5% of the Korean sub-sample kept medicine from their home country, compared to 59.7% for the Chinese sub-sample. Travel-based THP was more prevalent in the Chinese sample, with 74% who had traveled to China for healthcare compared to 38.1% for the Korean sub-sample.

Significant variables identified from Chi-square testing for Model 1 on non-travel-based THP included: demographic variables (group identity, year of immigration), local healthcare variables (frequency receiving quality primary care, primary language spoken with GP), spatial accessibility to SLGPs and a transnational tie variable (frequency contacting relatives from a participant’s home country). After entering these variables into Model 1, the results (see Table 2) indicated that group identity was the only significant independent variable predicting the likelihood of non-travel-based THP (*p* ≤ 0.05). Chinese older adult immigrants were 8.4 times more likely to access medicine from their home country compared to their Korean counterparts. Other demographics, local healthcare and transnational variables were non-significant in Model 1.

Model 2 explored the factors associated with travel-based THP. The independent variables included two demographic variables on group identity and year of immigration, a variable measuring geographic accessibility to a SLGP within the Toronto CMA, and a variable on transnational ties (i.e., financial assets) with the home country. Model 2 results (see Table 3) reveal that group identity and having financial assets in a participant’s home country were significant predictors of travel-based THP. More specifically, Chinese older adult immigrants were 8 times more likely to travel to China for healthcare, compared to the Korean older adult immigrants travelling to South Korea for healthcare. Participants with financial assets in their home country were approximately 18.4 times more likely to use travel-based THP.

### 6.2. Qualitative Analysis Results from Focus Groups

The FG participants’ demographic and THP patterns were similar to those of the broader survey. Demographics included higher counts of women, ages 65–84 and both ethnic groups engaged in non-travel-based and travel-based THP, with both being more prevalent among the Chinese FG. Thematic analysis of FGs revealed key barriers related to local healthcare access in Canada that impacted THP decisions in various ways. These barriers were related to wait times, language, cost, lack of culturally appropriate care, and poor spatial access to SLGPs. Participants who sought home country-based healthcare services reported experiencing shorter wait times, better communication with healthcare workers, sometimes cheaper healthcare options, and culturally preferred healthcare services through THP. Some participants expressed satisfaction with the Canadian healthcare system and their GPs, while still seeking THP due to individual and cultural preferences.

#### 6.2.1. Wait Time Barriers and THP

Participants in both groups discussed shorter wait times and better convenience associated with travel-based THP for primary and specialist care. Extensive wait times in Canada were overwhelmingly suggested by the FG participants as a significant barrier to quality healthcare. Common experiences with wait times included: lengthy wait times in waiting rooms of GPs and emergency department rooms and not receiving timely specialist appointments or surgeries. Participants who experienced long wait times locally, reported that seeing a doctor in their home country was faster and often resulted in immediate test results. As mentioned by Chinese participant (AP2), “no matter how good the specialist [in Canada] is, you can’t get timely treatment. It really worsens your condition,” in contrast seeking specialist care in China is a much faster and flexible process where patients could “choose by yourself which specialist […] appointment you want”. Similarly, Korean participants reported shorter wait times and improved accessibility to care in South Korea. According to Korean participant (KP75), “there’s a waiting period that’s far too long [in Canada], especially for specialist appointments […] Why does it take so long here, unlike [in] Korea?,” and when visiting Korea, seeing a doctor “[…] felt as easy as visiting a convenience store.”

#### 6.2.2. Communication and Language Barriers and THP

Language barriers faced by most of the Chinese and Korean participants led to strong preferences for non-travel-based and travel-based THP. Some participants relied on their family members for translation when visiting Canadian healthcare providers. Chinese participant BP6 mentioned that “[…] I couldn’t talk to people about my eyes because of the language barrier. Even if my daughter translates, she is not a medical student, so it is difficult for her to translate. So I especially wanted to go back to my country to have my eyes looked at.” Korean participant KP56 accounted a similar language-based barrier, reporting that “It’s so inconvenient, and when I go to the walk-in clinic, the doctor isn’t Korean. So I have to talk in English, so sometimes I write [my symptoms] at home [to show the doctor at the appointment]”. KP56 also reported travelling to Korea for a surgery and explained that “The reason I went to Korea is that I can communicate in the language […]”. Participants who received prescriptions written in English also reported difficulties with understanding medication instructions written in English. Chinese participant BP3 reported that when returning to Canada after visiting China, “[…] I always bring back some medicine. At least at one point, I can read the instructions, right? The medications prescribed in hospitals here are all in English.”

#### 6.2.3. Expense, Cost, Economic Barriers and THP

High costs for medications and healthcare services not covered by the OHIP is another main reason of seeking THP. When discussing certain high-cost services, such as dental procedures not covered by the Canadian healthcare system, Chinese participant DP2 reported that “Teeth are too expensive here [in Canada]”. Several Korean participants also reported dental care in Korea being more cost effective. According to KP54, “I do dental treatment or clinics, etc., when I go to Korea. I don’t do it here [in Canada], why? It’s too expensive […].” A small number of participants who had medical insurance in China or South Korea, obtained through a family member, former employer or other channels, used TPH strategically to reduce healthcare expenses. KP83 reported that dental implants were “significantly cheaper if you have insurance in Korea,” and KP102 reported traveling to Korean for dental and prostrate treatments because their medical insurance covers the expenses. However, most participants did not mention having access to health insurance in China or South Korea. A few mentioned that medical procedures and prescription medications covered by OHIP were more affordable in Canada, such as KP102 who says, “Without this [Canadian healthcare] system, I wouldn’t be able to afford it [medications]”. The appeal of cost-effective THP is then dependent on the procedure, type of medication desired and individual circumstances.

#### 6.2.4. Spatial Accessibility, Convenience Barriers and THP

FG participants who were engaged in THP also mentioned local healthcare access barriers related to convenience and ability to access care, including spatial accessibility and availability of doctors and transportation. Participants who had to travel long distances, e.g., Chinese participant BP6 who must “walk for 40 min”, to see their GP, indicates another layer of inconvenience in accessing local healthcare. This challenge was compounded for older adult immigrants with declining mobility and/or no car ownership. Participants also may rely on family members to transport them to doctor appointments, creating an additional barrier to seeing their GPs. Chinese participant BP4 said that “[…] you need to drive and communicate with the doctor. All of these require someone from my family.” BP4 was also found to use non-travel-based THP, reporting bringing back medicine from China and seeking Chinese traditional medicine in Canada.

### 6.3. Mixed-Methods Analysis: Integrating Quantitative and Qualitative Results

#### 6.3.1. Summary of Results

Cross-tabulation analysis was conducted to link key predictors of THP (i.e., significant variables from logistic regression models) and qualitative findings (i.e., FG quotes) by FG participants. A subset of the cross-tabulation approach, Table 4, shows charted responses from 5 Chinese and 5 Korean participants with diverse age and gender groups, who had a mix of travel-based and non-travel-based THP utilization (survey), reported different frequencies receiving quality primary care in Canada pre-COVID (survey), had/did not have financial assets in home country (survey), and shared their accounts in seeking local and transnational care (FGs). Results reveal that despite Chinese participants using both forms of THP more frequently than Korean participants, healthcare experiences, reasons and patterns for use of THP are similar.

#### 6.3.2. Non-Travel-Based THP

Non-travel-based THP (i.e., seeking home country-based medicine) existed in both study groups, but as revealed in Model 1, Chinese participants were 8.4 times more likely to use medicine from their home country compared to Korean participants. This difference was evidenced in the FGs, where Chinese participants discussed receiving medicine from China, ordering medicine online or bringing medicine back from China more often than their Korean participants. However, participants from the Korean group who did seek out medicine from their home country reported similar patterns of attaining medicine, such as through friends or family. For example, Korean participant KP83, describes a preference for a Korean medicine and said that “[…] whenever someone goes to Korea, I ask them to bring some for me”. Chinese participant DP2 also describes that “[…] when I go back to China to visit my family, I always bring some Chinese patent medicines”. Some Chinese and Korean participants also described seeking out home country-based medicine (e.g., herbal-based, over the counter medications, Chinese Traditional Medicine (TCM)) in Canada, as an alternative to transporting medicine to Canada. Chinese participant EP5 mentions, “We can buy prepared Chinese traditional medication here and you don’t need to travel back to China for that.” Some Korean participants reported use of TCM such as KP66 who “tried acupuncture and taken [Chinese] herbal medicine”. TCM is long rooted in Korean culture and shares many commonalities to Korean traditional medicine [54]. Discussion among Chinese participants reported TCM seeming less effective in Canada compared to China, leading to a preference of some participants to access medicine directly from China.

#### 6.3.3. Travel-Based THP

Travel-based THP is more prevalent than non-travel-based THP in both study groups, with Chinese participants engaged in travel-based THP more frequently. Model 2 revealed two significant variables predicting travel-based THP–membership with the Chinese group and having financial assets in home-country. Such quantitative patterns were compared to FG discussions in Table 4. While FG discussions on travel-based THP in Chinese groups were more extensive than Korean FGs, reasons and type of care sought between the groups were similar. Chinese participant BP5 and Korean participant KP75 both describe seeking travel-based THP as convenient, while participants BP6, DP4, KP54 and KP63 all report receiving dental care in home countries due to lower costs. Personal financial assets, due to its private nature, were not discussed widely in a group setting in FGs, but the on-line survey was deemed an appropriate platform for collecting such information. Model 2 indicates having financial assets a strong predictor of travel-based THP. FGs further revealed home country-based dental treatment as the most prevalent service sought by all the participants who had financial assets in their home countries and engaged in travel-based THP, as BP6 mentioned “dental care is so expensive here [in Canada].”

While local health care-related variables were not significant in Model 2, they are important to contextualize quantitative findings of the THP patterns, as supported by FG discussions on dissatisfaction with aspects of the Canadian healthcare system which led to decisions around use of THP. For example, participants from both ethnic groups who reported “often” receiving quality primary local healthcare, also indicated barriers faced in the Canadian healthcare system, and potential remediation to these barriers through THP. These trends are apparent in Table 4, such as Chinese participant BP5 who answered ‘often’ receiving quality local healthcare but also reported feeling dissatisfied with long wait times by saying that when visiting a doctor, “[…] you might show up at 2 o’clock and wait until 4 o’clock to be seen.” Subsequently, BP5 reported that shorter wait times and convenience were primary reasons for seeking THP. Similar experiences were found with Korean participants, such as KP75 who also answered ‘often’ receiving quality local primary healthcare but was dissatisfied with other forms of healthcare in Canada, specifying “a waiting period that’s far too long, especially for specialist appointments […]”. Certain participants who used both forms of THP, such as DP4, describe care in Canada as “[…] better than China.” These examples indicate that most participants, while experiencing numerous challenges in accessing care, tended to rate high in quality of care received in Canada. This is likely due to the subjective nature of the question on perception of local healthcare and partly explains the non-significant result of the variable, ‘frequency of receiving quality healthcare’ in Canada in Model 2.

## 7. Discussion

Through FGs and surveys, the study analyzed patterns of THP among older adult Chinese and Korean immigrants and associated demographic, local healthcare and transnational factors. Both groups consist of active users of travel-based and non-travel-based THP, with the Chinese group demonstrating a higher prevalence of THP. Consistent with the previous literature, the two groups are considered transnational migrants, who keep connections to their home countries through varying economic, social, and cultural ties; such as keeping financial assets in their home country [11,12,13,22,43,44]. Transnational connections, along with their culture preferences and the barriers in accessing local healthcare, shape the groups THP. This is supported by other studies of THP, indicating that transnationalism influences healthcare knowledge, attitudes, access and outcomes among transnational migrants [13,16,27].

Many Chinese and Korean participants who used THP navigated their health needs by mixing local healthcare with transnational healthcare usage; shortcomings in the Canadian healthcare system, especially related to specialty care, could be avoided or mitigated through THP. Most participants felt the quality of primary care received in Canada was satisfactory, despite reporting barriers of long wait times, and trouble communicating with English speaking GPs–the two barriers frequently brought up by both groups as reasons for seeking THP. Greater dissatisfaction was discussed by FGs surrounding specialty care not covered by OHIP, such as dental care, which was sought out in home-countries by participants who used travel-based THP.

The conjunction of local healthcare usage and THP has been documented for other transnational migrants [18,55,56,57]. Older adult immigrants who address their healthcare needs between healthcare systems in origin and destination countries suggest health inequities and their inability to receive quality healthcare and resources in one country [18]. This parallel healthcare usage can create risks for immigrants who access healthcare services between two countries, including a disruption of continuity of care, a lack of follow-up appointments to healthcare services and a risk of double treatment or overmedication [56,57]. Improved policy responses such as documentation on cross-border care and transnational frameworks for receiving healthcare in countries outside of Canada would help allow for continuous care between countries.

Even though the spatial access variable was found non-significant in logistic regression (Model 1) as a quantitative predictor of THP, in FGs poor spatial access to culturally competent healthcare in Canada emerged as an important aspect shaping older adult immigrants’ local and transnational healthcare experiences. Our results are consistent with findings from the literature [4,13,17,20], citing limited availability of same-language physicians in participants’ residential neighbourhood, a lack of access to proper transportation, long travel distance to GPs and declining mobility and disability, all of which are intertwined with other non-spatial factors such as cost, insurance and long wait time in driving THP for both study groups. For example, Wang and Kwak [13] reported health inequities for Korean older adult immigrants in Canada who were engaged in THP and experienced poor geographic access to the already limited Korean-speaking GPs, communication challenges, excessive wait time for procedures, and poor health status associated with old age.

The logistic regression (Model 2) reveals having financial assets in the home country, an economic and social aspect of transnationalism, as a significant predictor of travel-based THP. Transnational property ownership may be used as a family home for relatives, a possible place to return to, a holiday home, or to rent out [58]. As revealed in FGs, participants who accessed THP often maintained social connections to their home country, such as keeping in contact with family members and accessing healthcare while visiting them. Non-travel-based THP was also linked to social transnational ties, such as family members bringing medicine to participants while visiting in Canada. Other studies report that immigrants who use THP often maintain familial or social ties to their home countries [20,31,55].

Descriptive survey statistics indicated demographic differences among the two study groups, despite demographic variables (e.g., immigration year) not being significant in both Models 1 and 2. Chinese participants were more recent (immigrating after 2000) compared to Korean participants, who had mainly immigrated before 2000. Recent immigrants have been recorded to experience the greatest barriers when navigating healthcare in destination countries compared to long-standing immigrants [8,9], in particular, economic barriers [8]. Economic disparities can be exemplified in older immigrant populations of retirement age or those unable to work [42]. The majority of Chinese participants reported earning under $30,000 a year, compared to Korean participants who had lived in Canada longer and had a higher average income ($30,000–$49,999). Despite differences in economic status, both Chinese and Korean participants discussed lower costs as a potential benefit to travel-based THP, specifically dental care. Certain services such as dental and vision care are not covered and are costly without additional insurance, and as a result Chinese and Korean immigrants seek dental and eye care in their home countries which is more affordable than Canada [12,13]. One study revealed over a 4-year period 13% of immigrants in Canada sought dental care outside of Canada, often attributed to no dental insurance prominent among immigrants ages 50 and older [59]. Cost-effectiveness poses unique challenges to Chinese and Korean immigrants in Canada, as there can be high costs relating to air travel, privatized medical care, accommodations, and after-care [13]. Participants discussed follow-up care as a potential downside to travel-based THP, as they would need to stay in their home-country for an extended period to receive full treatment, resulting in greater costs. Receiving timely treatments, being able to communicate with healthcare workers comfortably, and experiencing culturally familiar practices, can outweigh the downside of travel-costs.

## 8. Limitations, Future Research, and Conclusions

Limitations in this study are related to primary data collection among older adult immigrants, particularly during the COVID-19 pandemic which introduced challenges and may lead to potential biases in data collection. Chinese older adult immigrants who filled in the online and telephone surveys and attended virtual focus groups on Zoom due to COVID-19 social distancing restrictions were required to have some level of technology proficiency. This may have excluded those without a digital device, digital literacy or technology support. Chinese participants were also recruited using a snowball sampling technique, which could have resulted in participant clusters within certain areas. Korean older adult immigrants were recruited through community organizations serving the Korean community (e.g., KCWA). They participated in the in-person surveys and focus groups, which required some level of mobility and having access to transportation. Sampling strategies could lead to possible overestimates of travel-based THP, since participants would be of higher socioeconomic backgrounds due to recruitment, self-selection, and travel resources. Future research would benefit from a random survey design to achieve more reliable statistical results, although this requires greater resources to administer.

Another limitation is related to the relatively small sample size of the surveys. While the original sample began with 201 participants, the removal of participants who left key answers blank, reduced the number to 161. Chinese and Korean samples were combined to allow for a large enough sample size to perform logistic regression models. Both Model 1 and Model 2 had wide CI intervals, indicating a small sample size and low precision in the models [60]. In Model 1, the variable “in correspondence with friends or family from home-country” had an OR of 499 × 10^6^ and a CI of 0.000–0.000, suggesting a perfect or quasi-complete separation which leads to unstable maximum likelihood estimates and is another indicator of the small sample size. Several important questions (e.g., other forms of non-travel-based THP, quality of specialist healthcare, and type of care sought from home country) were unable to be included in statistical analysis due to blank answers. This limited the assessment of other forms of non-travel-based THP, such as accessing health-related information online, an important THP format suggested by Troccoli et al. [21]. This study is a pilot study in understanding older adult immigrant’s THP. Building on this study, future research can aim for a larger sample size with strategies (e.g., providing adequate time, introducing break to avoid fatigue during survey) to assist older adult immigrants to improve completion and quality of survey answers. A larger number of participants would also help to meet minimum sample sizes for logistic regression analysis, and would produce more statistically accurate results that could apply to the larger Chinese and Korean populations in Canada.

To strengthen the analysis and to address the limitations of the small sample size, the mixed-methods approach combined quantitative and qualitative data analyses to identify overall group trends of THP and local healthcare patterns from surveys with the lived experiences and in-depth personal reasons for seeking THP among FG participants. Current research on THP generally employs quantitative and qualitative approaches separately, either based on small samples of immigrants to document their THP experiences through qualitative interviews or FGs [11,20,22,23,27,31], or uses quantitative surveys to highlight THP patterns [16,30,61]. This mixed-methods approach provides stronger inferences of the THP phenomenon and offers a more holistic picture of results, a benefit to mixed-methods research [62]. The spatial component of the analysis could be further advanced in future research to reflect more accurate spatial analysis. Such spatial access to physician measures could be further improved in future research by considering network distance and travel models.

The study provides new knowledge on THP in Canada and implications to inform health policy and healthcare for older adult immigrants. The findings highlight the necessity for the Canadian healthcare system to address systemic issues and improve care for older adult immigrants as their populations increase, especially relating to specialist care, wait times and cost-barriers. Barriers to quality local healthcare among immigrants influence decisions to seek non-travel-based and travel-based THP. Costs of privatized healthcare and travel to home countries, can potentially outweigh language, and systemic barriers to receive timely healthcare in Canada. As modern technology and travel advances, the exchange of medicine and health products, travel for healthcare across national borders, accompanied with Canadian healthcare issues may shape the future of healthcare, including THP, for older adult immigrant in Canada.

## Figures and Tables

**Table 1:** Demographic and THP of Survey Participants (*n* = 161).

Demographic Variable	Combined (%) (*n* = 161)	Chinese (%) (*n* = 77)	Korean (%) (*n* = 84)
Age			
55–64	25.4	29.9	21.4
65–74	34.2	33.8	34.5
75–84	39.8	36.3	42.9
≥85	0.6		1.2
Gender identity			
Women	67.7	71.4	64.3
Men	32.3	28.6	35.7
Household income			
Less than $30,000	43.5	58.4	29.8
$30,000 or above	56.5	41.6	70.2
Employment status			
Employed	14.9	15.6	14.3
Retired or non-working	85.1	84.4	85.7
Year of immigration			
Earlier than 2000	62.1	36.4	85.7
2001–2022	37.9	63.6	14.3
Non-Travel-based THP			
Yes	33.5	59.7	9.5
No	66.5	40.3	90.5
Travel-based THP			
Yes	55.3	74	38.1
No	44.7	26	61.9

**Table 2:** Model 1 results: Binary logistic regression for non-travel-based THP.

Independent Variable	OR	B	CI 95%	
			Lower Value	Upper Value
Group identity				
Chinese	8.417 *	2.130	2.699	26.247
Korean	0 ^a^			
Immigration year				
2001–2022	0.870	−0.139	0.356	2.126
2000 and earlier				
Language spoken most frequently with GP	0 ^a^			
Native-language or some native-language	1.474	0.388	0.526	4.131
English or other	0 ^a^			
Frequency of receiving quality healthcare pre-COVID				
Often	2.500	0.916	0.354	17.651
Sometimes	2.531	0.929	0.499	12.827
Less often	0 ^a^			
In correspondence with friends or family from home-country				
Yes	499 × 10^6^	20.030	0.000	0.000
No	0 ^a^			
Spatial accessibility to SLGP				
High	3.591	1.278	0.393	32.778
Average	3.123	1.139	0.323	30.218
Low	14.376	2.666	0.473	436.578
Very low	0 ^a^			

* *p* = 0.05. ^a^ = reference group.

**Table 3:** Model 2 results: Logistic regression for travel-based THP.

Independent Variable	OR	B	CI 95%	
			Lower Value	Upper Value
Group identity				
Chinese	8.065 *	2.088	3.097	21.006
Korean	0 ^a^			
Immigration year				
2001–2022	0.400	−0.917	0.146	1.090
2000 and earlier	0 ^a^			
Has a family doctor				
Yes	6.904	1.932	0.694	68.648
No	0 ^a^			
Has financial assets in home country (e.g., property ownership)				
Yes	18.478 *	2.917	3.522	96.935
No	0 ^a^			

* *p* = 0.05. ^a^ = reference group.

**Table 4:** Sample cross-tabulation linking quantitative (i.e., survey-based answers) and qualitative results (i.e., focus groups quotes).

Select Participant (Focus Group ID, Gender, Age)	Group Membership *	Frequency Receiving Quality Primary Care Pre-COVID (Survey)	Financial Assets in Home Country * (Survey)	Non-Travel-Based THP: Use Medicine Brought from Home Country (Survey)	Travel-Based THP Travel to Home Country for Health Services (Survey)	Quotes on Local Healthcare Experience (FGs)	Quote on THP (FGs)
BP6, Woman, 75–84	Mainland Chinese	Less Often	Yes	Yes	Yes	“I couldn’t talk to people about my eyes because of the language barrier. Even if my daughter translates, she is not a medical student […]”	“[In response to question about medical services sought in China] Mainly dental care. Because dental care is so expensive here (laughs).”
DP4, Man, 75–84	Mainland Chinese	Often	Yes	Yes	Yes	“I feel that the medical conditions here [in Canada] and the equipment for examination are quite good […] here is better than China”	“I do go to the dentist [in China][…] the dentist here [referring to Canada] is more expensive.”
EP5, Woman, 75–84	Mainland Chinese	Often	No	No	Yes	“I am very happy with my family doctors, they all speak Mandarin.”	“[…] I usually got new glasses from China. In addition, I sought dental care in China because my teeth were not good.”
BP5, Woman, 75–84	Mainland Chinese	Often	No	Yes	Yes	“[…] If you show up at the time they booked, but then… you might show up at 2 o’clock and wait until 4 o’clock to be seen.”	“I feel that it is much more convenient to see a doctor in China than here […] In China, it’s a one-stop shop.”
EP1, Man, 65–74	Mainland Chinese	Often	No	Yes	Yes	“We do have public health care here but what consequences it brings to us [regarding long wait times for test results]”	“[…] you have to immediately travel back to China if you have a serious disease unexpectedly […] Even though you spend your money, you can know your problem […]”
KP63, Woman, 75–84	South Korean	Sometimes	Yes	No	Yes	“I can’t understand the language, so even after I explain something, I can’t understand what It means […]”	“As far as I know, 90% of people around me who are 5% older go to Korea and get dental treatment.”
KP77, Woman, 55–64	South Korean	Often	No	No	No	“it’s comfortable for me because I can communicate in Korean. However the distance to get to [the doctor] from here is considerable […] It was quite inconvenient to deal with crowded waiting rooms and lengthy waiting times.”	N/A
KP56, Woman, 65–74	South Korean	Sometimes	No	No	Yes	“It’s so inconvenient, and when I go to the walk-in clinic, the doctor isn’t Korean. So I have to talk in English, so sometimes I write at home […].”	“The reason I went to Korea is that I can communicate in the language […]”
KP54, Man, 85 years old or above	South Korean	Often	No	No	Yes	“The new family doctor is very happy. Good job now, easily satisfying, kind and friendly.”	“I do dental treatment or clinics, etc. when I go to Korea. I don’t do it here, why? It’s too expensive.”
KP75, Woman, 75–84	South Korean	Often	No	Yes	Yes	“The dental fees [in Canada] are exorbitant. Also there’s a waiting period that’s far too long, especially for specialist appointments […]”	“So I went to see a doctor. It felt as easy as visiting a convenience store.”

* = *significant variable in logistic regression models*.

## Data Availability

Data used in this study are confidential in nature and cannot be shared.

## Appendix A. Survey Answer Re-Categorization for Quantitative Analyses

**Original Survey Question**	**Original Survey Answer Categories**	**Original Survey Answer Categories Combined**	**Variable Name**	**Variable Categories for Analysis**
1.7 Which of the following groups do you identify with?	(1) South Korean, (2) Mainland Chinese	N/A	Group identity	(1) South Korean, (2) Mainland Chinese
1.8 Can you tell us your 6-digit postal code AND nearest major intersection of your home?	Postal code and/or major intersection entered as a comment.	N/A. Spatial access to SLGP calculated based on residential postal code and postal code of SLGP.	Spatial access to same language physician	(1) High, (2) Average, (3) Low, (4) Very low
2.7 Please enter the first year you came to live in Canada:	Year entered as a comment.	Years combined to (1) 2000 and earlier, (2) 2001–2022	Year of immigration	(1) 2000 and Earlier, (2) 2001–2022
3.1 Do you have a family doctor?	(1) Yes, (2) No	N/A	Has a family doctor	(1) Yes, (2) No
3.7 Before the COVID-19 pandemic, when you needed primary health care services, how often did you receive quality care?	(1) All the time, (2) Most of the time, (3) Sometimes, (4) Rarely, (5) Never	Often as (1) All the time, (2) Most of the time; Less often as (3) Rarely, (5) Never	Frequency of receiving quality healthcare pre-COVID	(1) Often, (2) Sometimes, (3) Less often
4.2 How often do you correspond with friends or relatives in your home country?	(1) Everyday, (2) A few times a week, (3) Once a week, (4) Less than once a week, (5) Never	Yes as (1) Everyday, (2) A few times a week, (3) Once a week, (4) Less than once a week; No as (5) Never	In correspondence with friends or family from home-country	(1) Yes, (2) No
4.5 Do you have real estate or financial assets in your home country?	(1) Yes, (2) No	N/A	Financial assets in home-country	(1) Yes, (2) No
4.6 Do you keep medicine from your home country? (for example, brought to Canada in person, by others, or by mail)	(1) Yes, (2) No	N/A	Non-travel based THP	(1) Yes, (2) No
4.10 Since you came to Canada, have you travelled back to your home country and received healthcare services? (For example, healthcare services could include: general check-ups, traditional medicine, purchasing a prescription (glasses, medicine, etc.), seeing a doctor, surgeries.)	(1) Yes, (2) No	N/A	Travel-based THP	(1) Yes, (2) No
